# Potential for Person-to-Person Transmission of Henipaviruses: A Systematic Review of the Literature

**DOI:** 10.1093/infdis/jiad467

**Published:** 2023-10-31

**Authors:** Sonia T Hegde, Kyu Han Lee, Ashley Styczynski, Forrest K Jones, Isabella Gomes, Pritimoy Das, Emily S Gurley

**Affiliations:** Department of Epidemiology, Bloomberg School of Public Health, Johns Hopkins University, Baltimore, Maryland; Department of Epidemiology, Bloomberg School of Public Health, Johns Hopkins University, Baltimore, Maryland; Division of Infectious Diseases and Geographic Medicine, Stanford University, California; Department of Epidemiology, Bloomberg School of Public Health, Johns Hopkins University, Baltimore, Maryland; Department of Epidemiology, Bloomberg School of Public Health, Johns Hopkins University, Baltimore, Maryland; Institute of Health and Wellbeing, Federation University Australia, Ballarat, Victoria, Australia; Department of Epidemiology, Bloomberg School of Public Health, Johns Hopkins University, Baltimore, Maryland

**Keywords:** *Henipavirus*, Zoonosis, Person-to-person transmission, Systematic review

## Abstract

Nipah virus Bangladesh (NiV_B_) is a bat-borne zoonosis transmitted between people through the respiratory route. The risk posed by related henipaviruses, including Hendra virus (HeV) and Nipah virus Malaysia (NiV_M_), is less clear. We conducted a broad search of the literature encompassing both human infections and animal models to synthesize evidence about potential for person-to-person spread. More than 600 human infections have been reported in the literature, but information on viral shedding was only available for 40 case-patients. There is substantial evidence demonstrating person-to-person transmission of NiV_B_, and some evidence for NiV_M_. Less direct evidence is available about the risk for person-to-person transmission of HeV, but animals infected with HeV shed more virus in the respiratory tract than those infected with NiV_M_, suggesting potential for transmission. As the group of known henipaviruses continues to grow, shared protocols for conducting and reporting from human investigations and animal experiments are urgently needed.

## Teaser

Our systematic review of epidemiologic and animal laboratory studies highlights that, while the Bangladesh strain of Nipah virus is consistently transmitted between people, it is plausible that the Malaysian strain of Nipah virus and Hendra virus have similar transmission potential.

Zoonotic pathogens with demonstrated ability to infect and transmit between people are potential pandemic threats. In 2015, the World Health Organization (WHO) named Nipah virus (NiV) as one of the most dangerous emerging zoonotic disease threats because of its high case fatality and ability to transmit from person to person [1]. NiV belongs to the genus *Henipavirus*, in the family Paramyxoviridae, along with Hendra virus (HeV), which has caused spillovers into horses in Australia and has also caused human infections with severe clinical outcomes [2, 3]. The natural reservoirs for HeV and NiV include pteropodid bats, which are large fruit bats whose habitats span from South and Southeast Asia to East Africa and Australia.

Nipah virus outbreaks in Bangladesh and India have been reported since 2001 and have regularly been associated with person-to-person transmission [1, 2]. Evidence suggests that transmission occurs through close contact with patients and their respiratory secretions [1]. However, person-to-person transmission was not noted during the first outbreak identified in Malaysia and Singapore in 1998 and 1999 [2, 4], where spillover into pigs and transmission to abattoir workers were salient features. While very similar, the NiV strain identified in Bangladesh and India differed phylogenetically from the virus strain identified in the Malaysia and Singapore outbreak [5]. HeV has not been associated with person-to-person transmission and is distinct from NiV; partial N gene fragments can be used to genotype viruses at the major clade level (ie, HeV vs NiV Malaysia vs NiV India/Bangladesh) but cannot mimic full-genome genetic variation [5]. Epidemiologic evidence about person-to-person transmission across countries has led to the suggestion that differences in transmissibility might be driven by genetic differences in *Henipavirus* strains [6] and that the NiV India/Bangladesh strain is better adapted to human spread than other henipaviruses. However, an outbreak of *Henipavirus* in the Philippines in 2015 was associated with person-to-person transmission, and the virus implicated in the outbreak was more closely related to the Malaysian NiV strain than the South Asian strain [7].

Understanding which henipaviruses have the greatest potential for person-to-person spread would improve our scientific understanding of their pandemic risk. We conducted a systematic review and meta-analysis of human epidemiologic and clinical studies and studies of infections in mammals to compare transmission potential among the 3 henipaviruses known to infect humans: the India/Bangladesh strain of Nipah (NiV_B_), the Malaysian strain of Nipah (NiV_M_), and HeV.

## METHODS

### Literature Search and Study Selection

We conducted a broad search of the literature using PubMed, Embase, Cochrane Central Register of Controlled Trials, Web of Science, and Scopus. Gray literature was searched using IndMED, KoreaMED, and WHO Global Index Medicus. Search strategies (Supplementary Table 1) were used to identify publications indexed by 30 May 2019 using the genus (“*Henipavirus*”) and species names (“Hendra” and “Nipah”).

### Data Extraction and Analysis

For human studies, we extracted case-patient (or confirmed case) data including demographics, incubation period, symptomatology, whether or not the case was infected through person-to-person transmission, and clinical outcome. When available, we also extracted data on viral shedding by day post–illness onset. Our analysis summarized all available evidence about person-to-person transmission from extracted studies (Supplementary Appendix).

Information about viral shedding in humans was very rare, so we relied primarily on animal studies to characterize and compare viral shedding. For experimental animal studies, we extracted data on animal species, route and dose of inoculation, and viral shedding by type of biological specimen collected and day postinoculation. For observational studies of naturally infected animals, we extracted any information about viral shedding and type of biological specimen tested. We identified the studies that used the same animal model, dose, and comparable route of inoculation but with different *Henipavirus* strains and analyzed the results to compare key characteristics of transmission potential, including quantity and duration of viral shedding and symptoms (Supplementary Appendix).

## RESULTS

We identified 2465 studies that met our inclusion criteria for review (Supplementary Appendix, Supplementary Figure 1). After review, 52 human and 78 animal studies met the inclusion criteria for data extraction. The number of published studies increased over time, primarily among animal studies (Supplementary Appendix, Supplementary Figure 2).

### Human Studies

We identified 52 studies with data on confirmed human *Henipavirus* infections; 6 on HeV in Australia and the remaining 46 on NiV: 14 from Malaysia, 3 from Singapore, 1 from the Philippines (identified as Nipah-like virus), 19 from Bangladesh, and 9 from India (Supplementary Table 2). Twenty-nine studies (56%) investigated person-to-person transmission and 19 (37%) investigated viral shedding in oral, urine, or semen samples. Eleven studies (21%) investigated both transmission and viral shedding, and all such studies were from Bangladesh, India, and the Philippines.

### Transmission Potential of HeV

Only 6 human cases of HeV infection have been reported in the published literature [8–10], though 7 total human cases of HeV infection have occurred since 1994 [11], and none of these studies explicitly investigated person-to-person transmission. The presumptive exposure route was through contact with a sick or dead horse infected with HeV. However, 1 case of HeV had exposure to both an infected horse and a confirmed human case approximately 4 days prior to illness onset, such that person-to-person transmission could not be ruled out [12]. The case in question developed symptoms 11 days after exposure to an infected horse (performed a nasal cavity lavage during the last 3 days of the horse's incubation period) and was exposed in the workplace to the index case during his incubation period and first 2 days of illness. The index case developed symptoms 9 days after exposure to the same infected horse during the same procedure and presented with symptoms to the clinic 4 days prior to the case in question [12]. Among the 6 cases, 50% had respiratory symptoms and 50% died (Table 1); the seventh HeV case also died [11]. Three HeV cases from the 2004 and 2008 HeV outbreaks had oral or nasal and urine specimens collected to look for viral shedding (a total of 10 samples among the 3 case-patients were tested). Two of the 3 cases had evidence of HeV RNA by polymerase chain reaction (PCR) within 15 days post–illness onset (1 patient from oral/nasal specimen and 1 patient from urine specimen), both of whom also had signs of respiratory distress; the third case only had 1 sample taken 365 days post–illness onset, which was negative. One case-patient had urine samples at days 23 and 30 post–illness onset that had evidence of HeV RNA by PCR. There was no investigation of asymptomatic infections among exposed humans, though there was an investigation and evidence of asymptomatic infection among exposed horses [9, 13].

**Table 1.** Natural History of Infection for Henipaviruses by Country of Outbreak Origin Using Data From Outbreaks Identified During the 2001–2018 Time Period

Natural History Parameters	Virus and Country					
	HeV	NiV_M_			NiV_B_	
	Australia	Singapore	Malaysia	Philippines^a^	Bangladesh	India
Proportion of individuals exposed with evidence of asymptomatic infection	Not investigated	0.7% (10/1469) [14]	0.3% (4/1412) [19]	Not investigated	0% (0/1863) [1]	1.1% (3/279)^b^ [16, 20, 21]
Total number of infections (asymptomatic + symptomatic cases identified)	6	22	269	17	248	97
Proportion of all infections that are symptomatic	100% (6/6)	55% (12/22)	98.5% (265/269)	100% (17/17)	100% (248/248)	97% (94/97)
Median incubation period, d (range)	7.5 (5–16)	Not investigated	Not investigated	8 (4–20)	9 (6–14) [22]	10 (6–18)
Proportion of case-patients with respiratory signs or symptoms^c^	50% (3/6)^d^	50% (6/12) [14, 23]	21% (26/126)^e^ [24, 25]	94% (16/17)	63% (152/243) [1]	67% (63/94)
Case fatality	50% (3/6)	0.08% (1/12)	40% (105/265) [25]	53% (9/17)	78% (193/248)	93% (26/28)^f^

Data are presented as % (no./No.) unless otherwise indicated. References are cited to indicate from where data were used to derive estimates for each metric.

Abbreviations: HeV, Hendra virus; NiV_B_, Nipah virus Bangladesh; NiV_M_, Nipah virus Malaysia.

^a^Includes cases with meningitis (1 case), severe influenza-like illness (ILI; 5 cases), and acute encephalitis syndrome requiring ventilation (11 cases) in the Philippines outbreak [7]. No patients with meningitis or ILI died. We assume case-patients with acute encephalitic syndrome experienced respiratory distress based on the article description.

^b^Two hundred seventy-nine serum samples (155 healthcare workers and 124 household and community contacts) tested after the 2018 India NiV outbreak. Of the 279 samples, 2 were immunoglobulin M (IgM) and immunoglobulin G NiV positive and 1 was only IgM positive against NiV. The estimated overall seroprevalence was 1.08% (95% confidence interval, .37–3.11) (Kumar et al [21]).

^c^Respiratory signs and symptoms include cough, respiratory distress, difficulty breathing, ILI, and encephalitic syndrome when mechanical ventilation use is indicated.

^d^Two case-patients developed ILI illness and another had signs of severe respiratory distress.

^e^Although investigated, no substantial respiratory symptoms were reported from Goh et al [24] (February–June 1999 outbreak), but 13 case-patients reported nonproductive cough, which we include here as respiratory signs. Thirteen case-patients (40% of a total of 94 case-patients) from Wong et al [25] (September 1998–April 1999 outbreak) reported cough/respiratory symptoms.

^f^Case-fatality rate (CFR) reported for those cases that have data on outcome. The Siliguri (India) 2001 outbreak had an overall CFR of 68% (45/66), rendering the CFR for all cases 76% (71/94).

### Transmission Potential of NiV_M_

Studies on the transmission potential of NiV_M_ were conducted in Malaysia, Singapore, and the Philippines, representing a total of 294 symptomatic human cases and 308 total infections (Table 1). Of the 18 studies on NiV_M_ in humans, 4 investigated person-to-person transmission. In Malaysia and Singapore, although no healthcare workers who cared for hospitalized outbreak-related patients or abattoir workers (as the outbreak occurred near pig farms) developed symptoms suggestive of NiV_M_ infection, they were investigated to identify evidence of immunoglobulin G (IgG) antibodies against NiV_M_ [4, 14]. Three nurses in Malaysia and 22 healthcare workers in Singapore [14] had IgG antibody responses, though none of them had neutralizing antibodies. Ten abattoir workers (0.7% [10/1469]) were identified as having asymptomatic infections; however, the exposure was assumed to be infected pigs rather than infected humans [14]. Investigators of these outbreaks did not investigate person-to-person transmission to family caregivers, and the report from Singapore specifically stated that transmission within households was not investigated because of concerns of inciting panic [14]. Furthermore, as the outbreak was primarily investigated retrospectively, investigations of person-to-person transmission within households or family contacts of patients would have been very logistically difficult to do. The only evidence reported of transmission in households during the outbreak in Malaysia came from a report of an episode of late-onset encephalitis in a woman who did not live in the outbreak area but who had traveled to the area during the outbreak to care for her aunt who was a Nipah case-patient. The woman was diagnosed with late-onset Nipah encephalitis 11 years after the initial outbreak [15].

**Figure 1.**
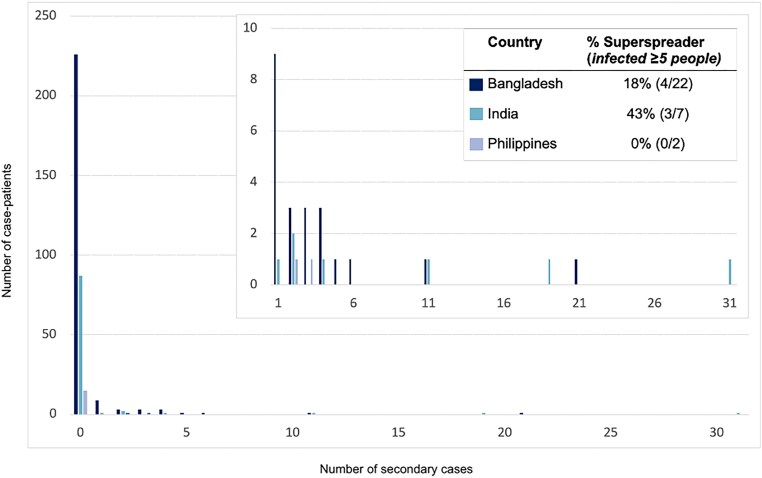
The distribution of secondary cases per Nipah case-patient (offspring distribution) and the proportion of those who transmitted who were superspreaders in countries where any person-to-person transmission of Henipaviruses has been observed—Bangladesh, India, and the Philippines, 2001–2018.

In the outbreak in the Philippines, 17 cases were identified. Seven cases slaughtered horses or consumed horse meat and 5 cases (29%) were exposed to other human cases but not to any horses. At least 12% (≥2/17) of case-patients infected 1 other person; based on history of patient contacts, 5 secondary cases were caused by person-to-person transmission from a minimum of 2 cases (Table 2; Figure 1).

**Table 2.** Description of Person-to-Person Henipavirus Transmission and Characteristics of Case-Patients Who Infected Others by Henipavirus Strain and Country in the Subset of Studies Where Person-to-Person Transmission Was Identified, 2001–2018

Case-patient Characteristics	Virus and Country		
	NiV_M_	NiV_B_	
	Philippines [7]^a^	Bangladesh [1]	India [16–18]
Proportion of case-patients who were transmitters (ie, infected someone else)	≥12% (≥2/17)	9% (22/248)	7% (7/94)
Proportion of transmitters who were male	100% (2/2)	77% (17/22)	75% (3/4)
Proportion of transmitters who were adults	100% (2/2)	77% (17/22)	100% (7/7)
Proportion of transmitters with respiratory symptoms	≥50% (≥1/2)	90% (20/22)	100% (4/4)
Proportion of secondary cases who were family members or nonprofessional caregivers	≥60% (3/5)	>45% (15/33)	≥54% (38/71)

Data are presented as % (no./No.). All estimates below are based on the studies where relevant information was provided.

Abbreviations: NiV_B_, Nipah virus Bangladesh; NiV_M_, Nipah virus Malaysia.

^a^Based on article description, we assumed at least 2 transmitters and that at least 1 transmitter had substantial respiratory secretions implying respiratory symptoms.

No patients infected with NiV_M_ had any samples collected to investigate viral shedding. While 23% (32/138) of patients identified in the outbreaks in Malaysia and Singapore had respiratory symptoms, 94% (16/17) of the patients in the Philippines outbreak did (Table 1).

### Transmission Potential of NiV_B_

All 28 studies of NiV_B_ outbreaks in Bangladesh and India, representing 342 total case-patients and 345 total infections (3 asymptomatic infections were found during a serological survey among patient contacts after the 2018 outbreak in India), have investigated the possibility of person-to-person transmission, and secondary cases have been regularly identified. Twenty-nine case-patients infected at least 1 other person (transmitter): 7% (7/94) of case-patients from India and 9% (22/248) of case-patients from Bangladesh (Table 2). Among the 29 NiV_B_ transmitters in the published literature, all were adults and 77% (20/26 where data was available) were male. Ninety-two percent of transmitters (24/26 with available data) had respiratory symptoms and all died.

Among case-patients who transmitted NiV_B_, 34% (10/29) transmitted to just 1 person and 24% (7/29) infected ≥5 other people; the size of outbreaks was heavily influenced by a few individuals who transmitted the virus to others as 8% of case-patients were responsible for the majority of transmission events (Figure 1). Roughly half (51%) of all 149 secondary cases were family members or nonprofessional caregivers and 9% were healthcare workers. In the 2018 India outbreak, during which detailed data were recorded, 22 of 23 total cases were a result of nosocomial transmission in 3 different hospitals [16]. The longest transmission chain reported was from a 2004 outbreak in Bangladesh, where 5 generations of transmission occurred [26].

**Figure 2.**
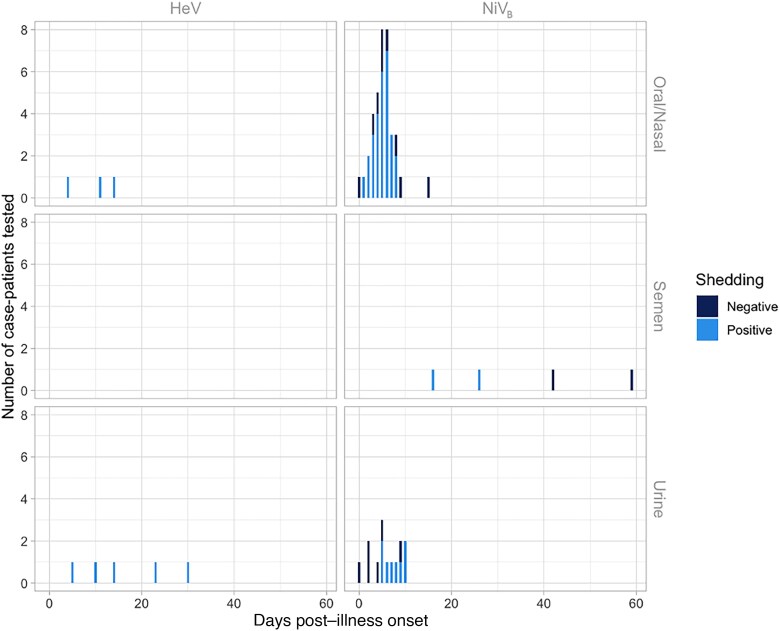
Evidence for *Henipavirus* viral shedding in 8 human oral/nasal and urine samples by days post–illness onset using polymerase chain reaction (PCR) testing for 3 unique case-patients with confirmed Hendra virus (HeV) infection from the 2004 and 2008 HeV outbreaks in Australia, and 55 human oral/nasal, semen, and urine samples by days post–illness onset using PCR testing for 37 unique case-patients with confirmed Nipah virus (NiV) infection from the 2008 and 2013–2014 Bangladesh outbreaks (NiV_B_) and 2001, 2007, and 2018 India NiV outbreaks. Though not shown in this figure, 2 additional urine samples from case-patients with HeV were PCR tested at days 365 (1 case-patient from the 2004 HeV outbreak) and 548 (1 case-patient from the 2008 HeV outbreak) post–illness onset. Both of these urine samples were PCR negative.

Seventeen patients from Bangladesh and 20 from India were investigated to identify viral shedding; 37 unique case-patients from the 2008 and 2013–2014 Bangladesh and 2001, 2007, and 2018 India NiV outbreaks provided 55 unique samples including urine, oral/nasal, and semen samples. One study from the 2018 NiV outbreak in India provided PCR cycle threshold (Ct) values to quantify viral load [27], and another study from Bangladesh [1] provided Ct values from throat swabs from case-patients, though no epidemiologic data accompanied these viral load data. There are 27 patients who contributed at least 1 oral or nasal swab sample in the published literature (total 37 samples), and 25 patients (total 32 samples) contributed samples during their first week of illness. Seventy-six percent (28/37) of respiratory samples that were tested had evidence of viral RNA, and 81% (26/32) had evidence of viral RNA in the first week of illness. Respiratory samples were collected an average of 7.6 days post–illness onset and samples that were positive were collected an average of 5.2 days after illness onset. More respiratory samples were collected 7 days post–illness onset than other days (n = 8) and all but 1 had evidence of NiV RNA by PCR (Figure 2). Only 1 patient was tested for viral RNA in semen and was found to be positive on days 16 and 26 but negative on days 42 and 59.

In Bangladesh, 1 study summarizing case findings from 14 years of data found that contacts of Nipah patients were significantly more likely to be infected if they had a longer duration of exposure to the patient (>12 hours), or if they had contact with the patient's body fluids, particularly respiratory secretions [1]. Contacts also reported close contact with Nipah case-patients toward the end of life in Bangladesh [28]. Anecdotal evidence from the Kerala, India outbreak in 2018 suggests a similar pattern where contact nearer to the day of death resulted in transmission more often than contact earlier in the course of illness [16].

For Nipah case-patients who died in Bangladesh and India and for whom we have information on time of symptom onset and death (n = 37), the median number of days from symptom onset to death was 6 (interquartile range [IQR], 5–7.5) for those who transmitted the virus to another person (mean, 6.7 [range: 3.5–13] days; n = 24) compared to 7 (IQR, 6–9.5) for case-patients who died but did not transmit (mean, 8.9 [range: 3–31] days; n = 39).

### Animal Studies

The route and dose of inoculation and types of biological samples collected varied substantially between study protocols, even within the same animal model, which made inferences about differences between viruses difficult (Supplementary Table 3). Given that person-to-person transmission is driven by exposure to respiratory secretions [1], we analyzed data from studies with PCR or reverse-transcription PCR results from oral, nasal, and/or nasopharyngeal samples that directly compared 2 or more *Henipavirus* strains within the same experiment or that only inoculated animals with 1 strain but used nearly identical methods with at least 1 other study (Supplementary Figure 3). Ultimately, among a total of 78 animal studies with primary data on viral shedding, we limited our analysis to 8 studies where the same methods were used for more than 1 virus within the same animal model to allow for equivalent comparisons: 4 African green monkey and 4 ferret studies. Results from ferret studies were insufficient in study design and sample numbers to draw any strong conclusions about differences between viruses (Supplementary Appendix, Supplementary Table 3).

The 4 African green monkey studies [29–32] included in the analysis were conducted by the same research group and used nearly identical protocols, though the route of inoculation differed slightly across studies (Mire et al [29] and Geisbert et al [30] inoculated intratracheally; Mire et al [31, 32] inoculated intranasally and intratracheally). In all, 20 animals were inoculated with 10^5^ plaque-forming units with 3 different *Henipavirus* strains: 7 with NiV_B_, 9 with NiV_M_, and 4 with HeV.

**Figure 3.**
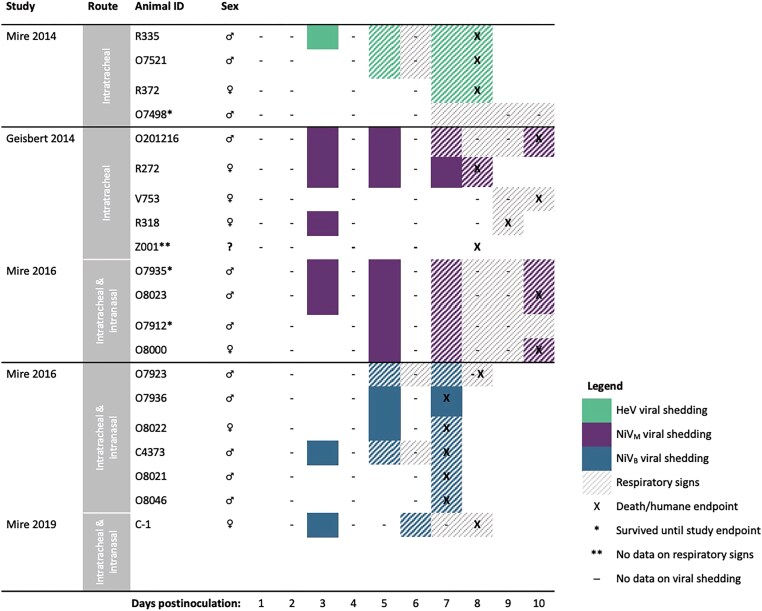
Comparing onset and duration of oral viral shedding and respiratory signs, and timing of humane endpoints among African green monkeys (n = 20) infected with Hendra virus (HeV), Nipah virus Bangladesh (NiV_B_), or Nipah virus Malaysia (NiV_M_), by study. The studies used here, including the route of inoculation employed, are as follows: Mire et al [29] (intratracheal; n = 4), Geisbert et al [30] (intratracheal; n = 5), Mire et al [31] (intratracheal and intranasal; n = 10), and Mire et al [32] (intratracheal and intranasal; n = 1).

All animals inoculated with HeV and more than half of the animals inoculated with NiV_M_ were first investigated for viral shedding 3 days postinoculation (Figure 3). One of 4 animals (25%) inoculated with HeV began shedding on day 3 postinoculation and 50% were shedding by day 5; 67% (6/9) of animals inoculated with NiV_M_ and 57% (4/7) of animals inoculated with NiV_B_ were shedding by day 5. Shedding often occurred prior to the onset of respiratory symptoms for animals with detectable virus (59% [10/17]) (Figure 3, Table 3). Viral shedding typically continued until the animal reached criteria for euthanasia (Figure 3, Table 3). Time to euthanasia was longer for African green monkeys inoculated with NiV_M_ (mean, 9.3 [range, 8–10] days) compared to NiV_B_ (mean, 7.3 [range, 7–8] days) and HeV (mean, 8.0 [range, 8–8] days), contributing to longer shedding durations in animals inoculated with NiV_M_ (mean, 4.8 [range, 0–8] days) compared to NiV_B_ (mean, 2.8 [range, 0–5] days) and HeV (mean, 3.0 [range, 0–6] days).

**Table 3.** Comparing the Trajectory of Infection, Including Timing of Viral Shedding, Respiratory Signs, and Euthanasia Among African Green Monkeys Infected With Hendra Virus, Nipah Virus Malaysia, or Nipah Virus Bangladesh in Studies That Used Similar Protocols (n = 20)

Time in Days	HeV	NiV_M_	NiV_B_
	Mean No. of Days (Range), no./No.		
Time from inoculation to:			
Respiratory signs, among animals with signs	6.0 (5–7) 4/4	7.6 (7–9) 8/9	6.2 (5–7) 6/7
Euthanasia, among animals euthanized	8.0 (8–8) 3/4	9.3 (8–10) 7/9	7.3 (7–8) 7/7
Oral shedding, among animals with shedding	5.0 (3–7) 3/4	3.6 (3–5) 7/9	5 (3–7) 7/7
Total oral shedding duration, among all animals^a^	3.0 (0–6) 4/4	4.4 (0–8) 9/9	3.0 (0–5) 7/7
Duration of oral shedding prior to respiratory signs or euthanasia, among animals with shedding	0.7 (0–2) 3/4	3.1 (1–5) 7/9	1.3 (0–3) 7/7

The sample size (no./No.) indicates how many animals provided data for the estimation of reported time in days.

Abbreviations: HeV, Hendra virus; NiV_B_, Nipah virus Bangladesh; NiV_M_, Nipah virus Malaysia.

^a^Endpoint was defined as the last day with a positive polymerase chain reaction result. Day of euthanasia was used instead for 1 HeV animal (O7923) not tested on the day of euthanasia.

African green monkeys inoculated with NiV_M_ developed respiratory signs later (mean, 7.6 [range, 7–9] days) than those inoculated with NiV_B_ (mean, 6.2 [range, 5–7] days) and HeV (mean, 6.0 [range, 5–7] days) (Figure 3, Table 3). This resulted in longer periods of viral shedding prior to the onset of respiratory signs or euthanasia in the case of animals without signs. Animals inoculated with NiV_M_ shed virus for an average of 3.1 (range, 1–5 days) prior to signs, compared to 1.3 (range, 0–3) days with NiV_B_ and 0.7 (range, 0–2) days with HeV.

**Figure 4.**
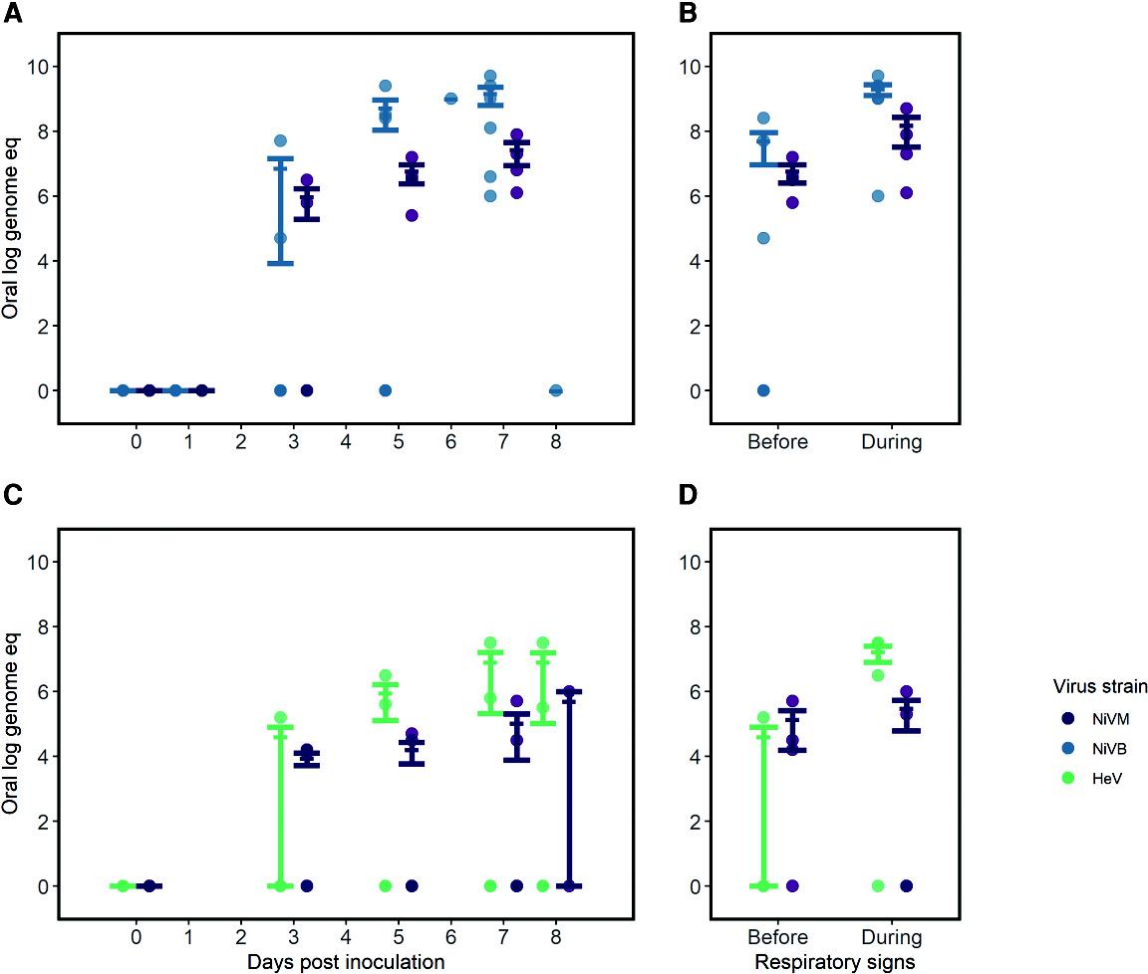
Comparing virus quantity in oral samples of African green monkeys infected with Hendra virus (HeV) or Nipah virus Bangladesh (NiV_B_) compared with Nipah virus Malaysia (NiV_M_) in studies with similar study protocols, by route of inoculation. *A* and *B*, Virus quantity by days postinoculation (*A*) and peak quantity before and during respiratory signs (*B*) in animals inoculated intratracheally with NiV_M_ and NiV_B_ [29, 30] (n = 9). *C* and *D*, Virus quantity by days postinoculation (*C*) and peak quantity before and during respiratory signs (*D*) in animals inoculated intranasally/intratracheally [31, 32] with NiV_M_ and HeV (n = 11). Quantities are shown as log genome equivalents. Error bars represent mean and standard error. Four African green monkeys were excluded from *B* and *D* as no samples were positive by polymerase chain reaction and/or the animals did not develop respiratory signs.

In oral samples, we observed a mean virus quantity over 100-fold higher starting on day 5 postinoculation in African green monkeys inoculated with NiV_B_ compared to NiV_M_ and HeV (Figure 4*A*). The same pattern was not observed in nasal samples, where we noted a similar rise in virus quantity for both NiV_B_ and NiV_M_ by day 1 postinoculation (Supplementary Figure 4). Among animals with HeV, we observed consistently lower virus quantities in nasal samples with 2 of 4 animals having no detectable virus up to 7 days postinoculation; however, all 4 animals inoculated with HeV were inoculated intratracheally, as opposed to both intratracheal and intranasal routes in other studies. For each virus strain, the mean virus quantity increased in oral samples after onset of respiratory symptoms (Figure 4*B* and 4*D*). Animals inoculated with NiV_B_ expressed the highest virus quantity in oral samples both before and during respiratory signs. There were no studies that reported on detection of culturable virus from oral or nasal specimens.

## DISCUSSION

Our knowledge about the diversity of zoonotic henipaviruses in nature continues to grow but remains limited. A new *Henipavirus* infecting shrews and humans, but with no evidence of person-to-person transmission, was reported from China in 2022 [33]. Named Langya virus, it is genetically more similar to Mòjiāng virus, which was previously identified in rodents, than to Nipah or Hendra. In 2021, a new Hendra virus was identified from fruit bats in Australia that had been previously missed by surveillance because the genetic differences in the virus made it undetectable by the PCR diagnostics routinely used [34]. Given the threat these viruses pose for human health, we should aim to understand as best we can the ecology of these viruses and the biological and social determinants of transmission and transmission potential.

There is substantially more evidence demonstrating the transmission potential of NiV_B_, compared to NiV_M_ or HeV, from both the studies of human epidemiology and animal infection. Two studies have demonstrated environmental contamination with viral RNA [26, 35], but the role of environmental contamination in transmission remains unknown. However, the role of viral shedding in urine or semen in contributing to onward transmission remains unexplored. If transmission between people is driven by the amount of virus shed in respiratory secretions and only a few case-patients transmit the virus to the majority of secondary cases, then we would expect high variation in the amount of virus shed by individuals as a major driver of human transmission potential. The substantial variation in the amount of virus shed between African green monkeys infected with NiV_B_ in respiratory secretions supports the conclusion that differences in the number of cases each person infects may be primarily driven by the amount of virus they shed.

Our review also suggests that NiV_M_ poses a risk for person-to-person spread, based on anecdotal reports from Malaysia and the Philippines outbreak [15]. Although nonhuman primates infected with NiV_M_ did not shed as much virus as those infected with NiV_B_, they were symptomatic and shed virus longer, which could also pose a transmission advantage. The potential for HeV to be transmitted between people is less clear but should not be discounted. With only 6 human cases of HeV reported in the literature and 7 human cases ever reported, there are too few observations to determine if there are real differences in the transmission potential in humans compared to NiV_B_ or NiV_M_. Animals infected with HeV shed significantly higher titers of virus in respiratory specimens than animals infected with NiV_M_ in the same study.

Twenty-seven years have passed since the first human infection with a *Henipavirus* was reported [13], yet among the >600 human infections reported in the literature, only 40 have had data investigating viral shedding reported. Many studies did not investigate transmission to household contacts [7, 14], and 1 outbreak in Siliguri, India, where nosocomial transmission was a key driver of the outbreak, provides too few details to re-create transmission chains or identify superspreading events [17]. Given that nosocomial spread has fueled many infectious disease outbreaks, understanding the details of transmission within healthcare settings is critical to designing effective containment and infection control strategies. Moreover, if we want to improve our understanding of the transmission potential of henipaviruses and distinguish the characteristics of these highly fatal viruses that might contribute to differential transmission dynamics, we need a systematic approach to human epidemiologic and clinical investigations and animal models. Standardized protocols to investigate person-to-person spread and biological and behavioral risk factors for transmission, including the potential for sexual transmission, across henipaviruses and settings would leverage opportunities to learn about these emerging viruses (Supplementary Appendix).

The published experimental studies we identified in our review were largely unsuitable for providing meaningful comparisons across studies because of differences in inoculation routes and titers, and frequency of and methods for measuring viremia and viral shedding, which did not differentiate viable from nonviable virus. These studies are costly and time consuming and we should maximize their ability to inform our understanding of transmission through greater harmonization of protocols across laboratories and animal models.

Though our review highlights NiV_B_ as a greater threat to humans compared to the other *Henipavirus* strains, we cannot dismiss the risk that the other known and yet undiscovered viruses may pose. As the group of known henipaviruses continues to grow, shared protocols for human investigations and animal experiments are urgently needed to capitalize on more opportunities to advance our understanding of transmission risk.

## Supplementary Data

Supplementary materials are available at *The Journal of Infectious Diseases* online (http://jid.oxfordjournals.org/). Supplementary materials consist of data provided by the author that are published to benefit the reader. The posted materials are not copyedited. The contents of all supplementary data are the sole responsibility of the authors. Questions or messages regarding errors should be addressed to the author.

## Supplementary Material

jiad467_Supplementary_Data

## Notes

***Author contributions*.** E. S. G. conceived the study idea. E. S. G. and K. L. worked with an informationist to design the search strategy. K. L., S. H., A. S., and F. K. J. performed the analyses. All authors reviewed studies for inclusion in the analysis, extracted data from studies, and contributed to manuscript writing.
