# Correction to: Potential for Person-to-Person Transmission of Henipaviruses: A Systematic Review of the Literature

**DOI:** 10.1093/infdis/jiaf487

**Published:** 2025-09-26

This is a correction to: Hegde ST, Lee KH, Styczynski A et al., Potential for Person-to-Person Transmission of Henipaviruses: A Systematic Review of the Literature, *The Journal of Infectious Diseases,* Volume 229, Issue 3, 15 March 2024, Pages 733–742, https://doi.org/10.1093/infdis/jiad467.

In Table 1 of the original publication of this article, the NiV-M Malaysia strain Proportion of individuals exposed with evidence of asymptomatic infection should be 0.3% (4/1412) and was erroneously reported to be 3% (4/1412).

