# Supplementary material for: Potential for Person-to-Person Transmission of Henipaviruses: A Systematic Review of the Literature
Source: J Infect Dis. 2023 Oct 31;229(3):733–42. doi: 10.1093/infdis/jiad467 (PMC10938219; doi:10.1093/infdis/jiad467)
Supplement: jiad467_Supplementary_Data [file jiad467_supplementary_data.docx]

**Supplementary Appendix**

**Additional literature search and review methods**

We reviewed the references of selected papers and identified 2 studies published prior to the use of ‘Hendra’, when it was referred to as ‘Equine morbillivirus’, that we included in the review, and 1 study on non-human primates which was published on May 29, 2019 but was not yet indexed.

Titles and abstracts were independently reviewed by two members of the study team and excluded if they lacked primary data, were an *in vitro* experiment, or were in languages other than English, French, Spanish, and Dutch. We also excluded studies of infection in bats as these do not provide evidence of potential human-to-human spread. Studies were included for data extraction if two team members reviewed the full text and confirmed that the study included human studies with primary data on secondary attack rates or transmission between people, or viral shedding (from any biological specimen) or viremia in either humans or experimentally infected mammals. Any discrepancies between reviewers were resolved through discussion between all reviewers.

**Additional data analysis methods**

*Human studies*

We calculated the proportion of case-patients that experienced respiratory symptoms, median incubation period, case-fatality, and secondary attack rates and compared these metrics between henipaviruses. We defined respiratory symptoms as those case-patients that either were explicitly described to have respiratory distress, difficulty breathing, or “influenza-like illness (ILI) and encephalitis”, as some articles described case-patients with acute encephalitic syndrome as experiencing respiratory distress. We described the number of secondary cases for all Nipah virus cases where this information was published. We graphed the proportion of patients who shed virus in respiratory secretions by days since symptom onset using available data.

*Animal studies*

As exposure to respiratory secretions is considered the primary route of person-to-person transmission[^1^](https://paperpile.com/c/8ecMQN/XH36), we focused our analysis on animal viral shedding from the respiratory tract. We examined the virus quantity (in genome equivalents) shed post inoculation in oral and nasal samples as a proxy measure of infectiousness for person-to-person transmission[^8,9^](https://paperpile.com/c/8ecMQN/10z7+Q6K6), and we examined the infectious period, as determined by polymerase chain reaction (PCR) or real-time polymerase chain reaction (RT-PCR) viral detection in the days post inoculation, as a proxy measure of the duration of viral shedding. We also examined the duration of viral shedding prior to the onset of respiratory signs and the peak viral quantity both before and during respiratory signs.

**Supplementary Figure 1.** Study selection


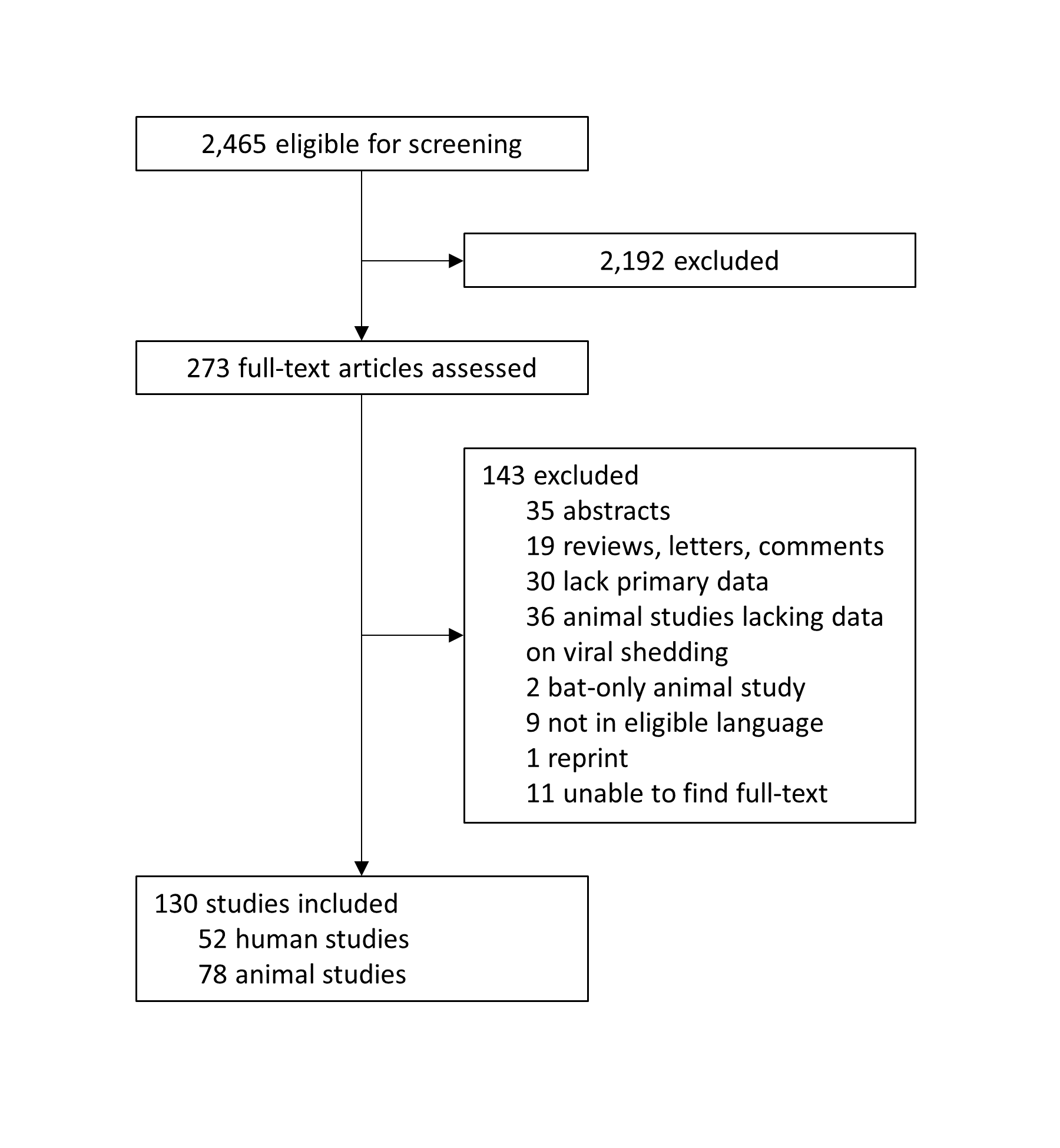


**Supplementary Figure 2.** Studies included in systematic review, by study type and year of publication.


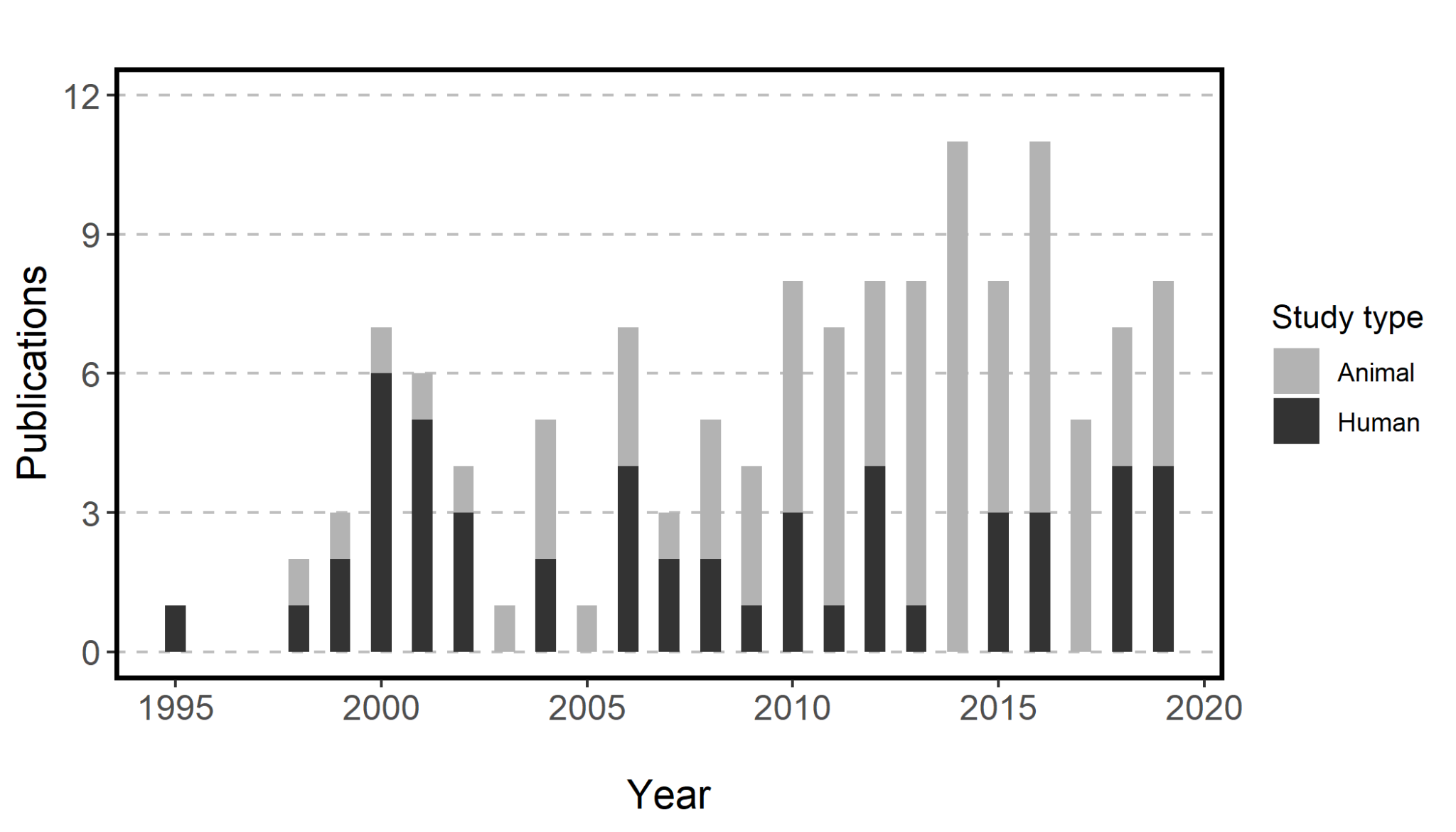


**Supplementary Figure 3.** Reasons for animal study exclusion.

**
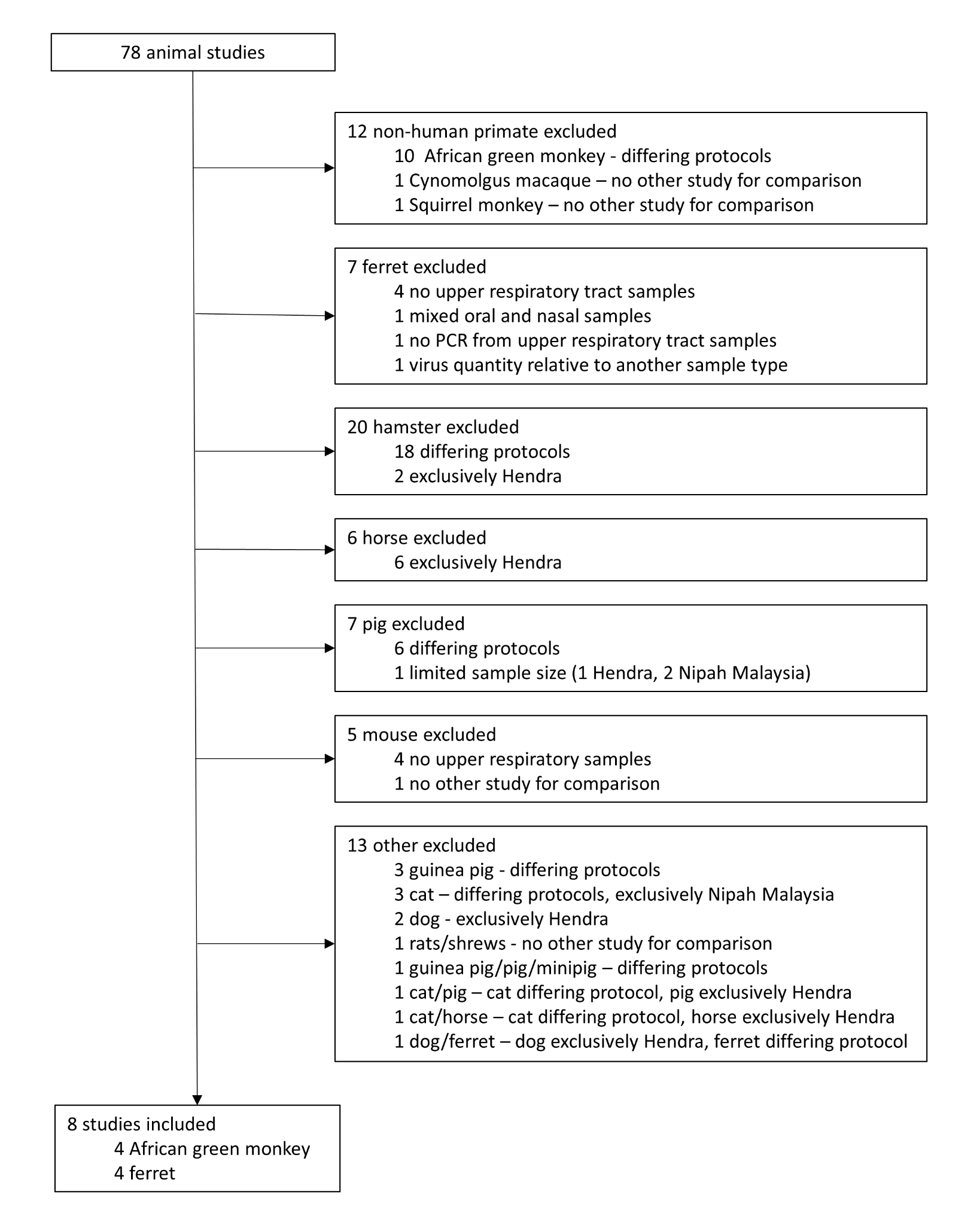
**

## **Supplementary Figure 4.** Virus quantity in nasal samples of African green monkeys inoculated in studies with similar study protocols. Quantities are shown as log genome equivalents by day post inoculation (A) and as the peak value before and during respiratory signs (B). Error bars represent mean and standard error. Four African green monkeys were excluded from B as no samples were positive by PCR and/or did not develop respiratory signs.

## **
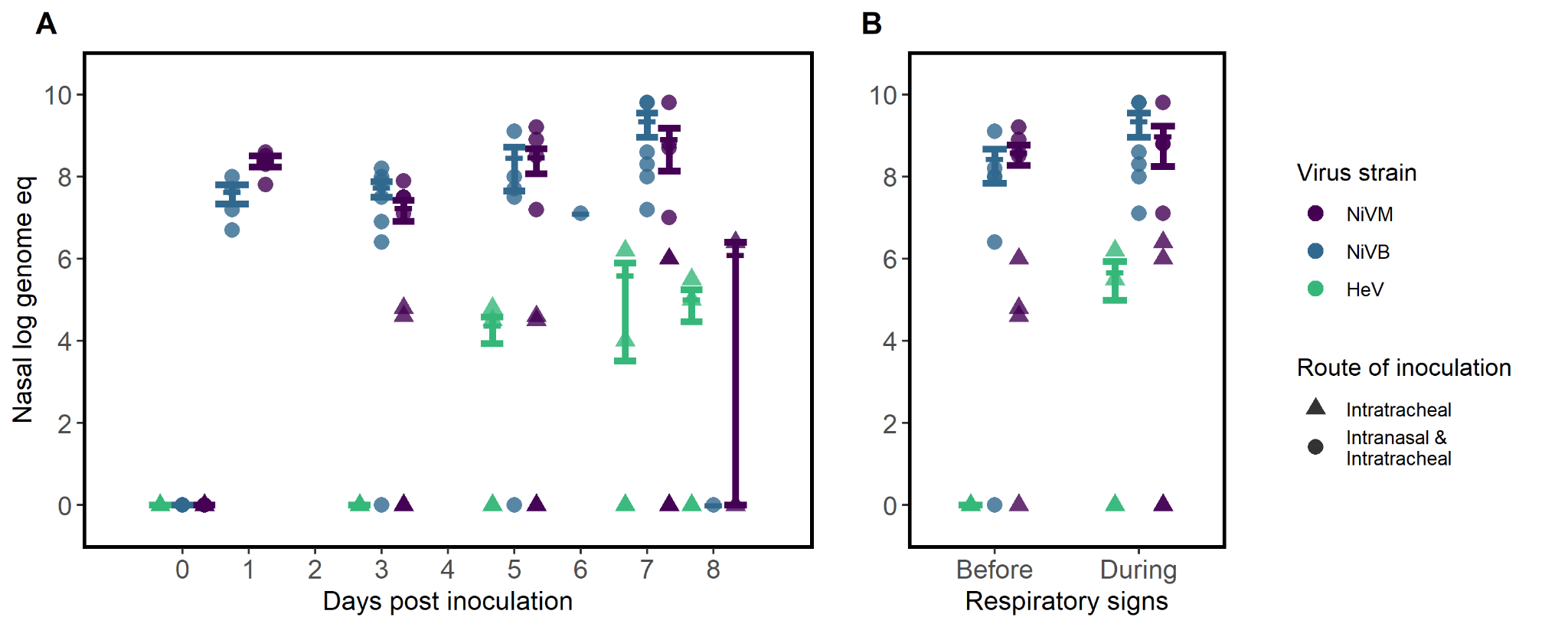
**

##

## **Supplementary Table 1. Electronic databases and search strategies**

| **Electronic database** | **Search strategy** |
| --- | --- |
| PubMed | ("Henipavirus"[Mesh] OR "Henipavirus Infections"[Mesh]) OR  (Henipa*[tw] OR hendra*[tw] OR nipah*[tw]) |
| Embase | ('henipavirus'/exp OR 'henipavirus infection'/exp) OR ((Henipa* OR hendra* OR nipah*):ti,ab,kw) |
| Cochrane Central Register of Controlled Trials | MeSH descriptor: [Henipavirus] explode all trees OR MeSH descriptor: [Henipavirus Infections] explode all trees OR (Henipa* OR hendra* OR nipah*) |
| Web of Science | TS=(Henipa* OR hendra* OR nipah*) |
| Scopus | TITLE-ABS-KEY: henipa* OR hendra* OR nipah* |
| IndMED | Henipa OR Henipavirus OR hendra OR hendra virus OR nipah OR nipah virus |
| KoreaMED | Henipa* OR hendra* OR nipah* |
| WHO Global Index Medicus | Henipa* OR Hendra* OR Nipah* OR MH:B04.820.455.600.650.400 OR MH:C02.782.580.600.400 OR MH:B04.820.455.600.650.400.400 OR MH:B04.820.455.600.650.400.550 |

## **Supplementary Table 2.** Studies published through May 2019 on human henipavirus infections, by year and country (N=52)

| **Virus** | **Country** | **Year** | **Study** | **Investigated person-to-person transmission** | **Investigated viral shedding** |
| --- | --- | --- | --- | --- | --- |
| Hendra | Australia | 1995 | Selvey LA, et al. Infection of humans and horses by a newly described morbillivirus  PMID: 7603375 | + | - |
| Hendra | Australia | 1998 | Paterson DL, et al. Zoonotic Disease in Australia Caused by a Novel Member of the Paramyxoviridae  PMID: 9675464 | + | - |
| Hendra | Australia | 2006 | Hanna JN, et al. Hendra virus infection in a veterinarian  PMID: 17371223 | - | - |
| Hendra | Australia | 2009 | Wong KT, et al. Human Hendra virus infection causes acute and relapsing encephalitis  PMID: 19473296 | - | - |
| Hendra | Australia | 2010 | Playford EG, et al. Human Hendra virus encephalitis associated with equine outbreak, Australia, 2008  PMID: 20113550 | - | + |
| Hendra | Australia | 2012 | Taylor C, et a. No evidence of prolonged Hendra virus shedding by 2 patients, Australia  PMID: 23171522 | - | + |
| Nipah | Malaysia | 1999 | Chua KB, et al. Fatal encephalitis due to Nipah virus among pig-farmers in Malaysia  PMID: 10520635 | - | - |
| Nipah | Malaysia | 2000 | Premalatha GD, et al. Assessment of Nipah Virus Transmission Among Pork Sellers in Seremban Malaysia  PMID: 11127331 | - | - |
| Nipah | Malaysia | 2000 | Parashar UD, et al. Case-Control Study of Risk Factors for Human Infection with a New Zoonotic Paramyxovirus, Nipah Virus, during a 1998–1999 Outbreak of Severe Encephalitis in Malaysia  PMID: 10823779 | - | - |
| Nipah | Malaysia | 2000 | Goh KJ, et al. Clinical features of Nipah virus encephalitis among pig farmers in Malaysia  PMID: 10781618 | - | + |
| Nipah | Malaysia | 2000 | Amal NM, et al. Risk factors for Nipah virus transmission, Port Dickson, Negeri Sembilan, Malaysia: results from a hospital-based case-control study  PMID: 11127330 | - | - |
| Nipah | Malaysia | 2000 | Chua KB, et al. High mortality in Nipah encephalitis is associated with presence of virus in cerebrospinal fluid  PMID: 11079547 | - | - |
| Nipah | Malaysia | 2001 | Wong SC, et al. Late presentation of Nipah virus encephalitis and kinetics of the humoral immune response  PMID: 11561048 | - | - |
| Nipah | Malaysia | 2001 | Chua KB, et al. The presence of Nipah virus in respiratory secretions and urine of patients during an outbreak of Nipah virus encephalitis in Malaysia  PMID: 11243752 | - | + |
| Nipah | Malaysia | 2001 | Sahani M, et al. Nipah virus infection among abattoir workers in Malaysia, 1998-1999  PMID: 11689513 | - | - |
| Nipah | Malaysia | 2001 | Mounts AW, et al. A cohort study of health care workers to assess nosocomial transmissibility of Nipah virus, Malaysia, 1999  PMID: 11181159 | + | - |
| Nipah | Malaysia | 2001 | Ali R, et al. Nipah virus among military personnel involved in pig culling during an outbreak of encephalitis in Malaysia, 1998-1999  PMID: 11592256 | - | - |
| Nipah | Malaysia | 2002 | Tan CT, et al. Relapsed and late-onset Nipah encephalitis  PMID: 12112075 | - | - |
| Nipah | Malaysia | 2002 | Wong KT, et al. Nipah virus infection: pathology and pathogenesis of an emerging paramyxoviral zoonosis  PMID: 12466131 | - | - |
| Nipah | Malaysia | 2012 | Abdullah S, et al. Late-onset Nipah virus encephalitis 11 years after the initial outbreak: A case report  PMID: NA  Journal: *Neurology Asia* | + | - |
| Nipah | Singapore | 1999 | Paton NI, et al. Outbreak of Nipah-virus infection among abattoir workers in Singapore  PMID: 10520634 | - | - |
| Nipah | Singapore | 2000 | Chew MHL, et al. Risk factors for Nipah virus infection among abattoir workers in Singapore  PMID: 10823780 | - | - |
| Nipah | Singapore | 2002 | Chan KP, et al. A survey of Nipah virus infection among various risk groups in Singapore  PMID: 11895096 | + | - |
| Nipah | Bangladesh | 2004 | Hsu V, et al. Nipah Virus Encephalitis Reemergence, Bangladesh  PMID: 15663842 | + | - |
| Nipah | Bangladesh | 2004 | WHO. Nipah virus outbreak(s) in Bangladesh, January-April 2004  PMID: 15132054 | + | - |
| Nipah | Bangladesh | 2006 | Luby SP, et al. Foodborne transmission of Nipah virus, Bangladesh  PMID: 17326940 | + | - |
| Nipah | Bangladesh | 2007 | Gurley ES, et al. Risk of nosocomial transmission of Nipah virus in a Bangladesh Hospital  PMID: 17520553 | + | - |
| Nipah | Bangladesh | 2007 | Gurley ES, et al. Person-to-person transmission of Nipah virus in a Bangladeshi community  PMID: 18214175 | + | + |
| Nipah | Bangladesh | 2008 | Hossain MJ, et al. Clinical presentation of Nipah virus infection in Bangladesh  PMID: 18444812 | - | - |
| Nipah | Bangladesh | 2008 | Montgomery JM, et al. Risk factors for Nipah virus encephalitis in Bangladesh  PMID: 18826814 | + | - |
| Nipah | Bangladesh | 2010 | Homaira N, et al. Cluster of Nipah Virus Infection, Kushtia District, Bangladesh, 2007  PMID: 21042407 | + | - |
| Nipah | Bangladesh | 2010 | Homaira N, et al. Nipah virus outbreak with person-to-person transmission in a district of Bangladesh, 2007  PMID: 20380769 | + | - |
| Nipah | Bangladesh | 2012 | Rahman MA, et al. Date palm sap linked to Nipah virus outbreak in Bangladesh, 2008  PMID: 21923274 | + | + |
| Nipah | Bangladesh | 2012 | Lo MK, et al. Characterization of Nipah virus from outbreaks in Bangladesh, 2008-2010  PMID: 22304936 | - | + |
| Nipah | Bangladesh | 2013 | Sazzad HMS et al. Nipah virus infection outbreak with nosocomial and corpse-to-human transmission, Bangladesh  PMID: 23347678 | + | + (overlapping study with Lo 2012) |
| Nipah | Bangladesh | 2015 | Sazzad HMS, et al. Exposure-Based Screening for Nipah Virus Encephalitis, Bangladesh  PMID: 25625615 | + | - |
| Nipah | Bangladesh | 2015 | Naser AM, et al. Integrated cluster- and case-based surveillance for detecting stage III zoonotic pathogens: an example of Nipah virus surveillance in Bangladesh.  PMID: 25342551 | + | - |
| Nipah | Bangladesh | 2016 | Islam MS, et al. Nipah virus transmission from bats to humans associated with drinking traditional liquor made from date palm sap, Bangladesh, 2011-2014  PMID: 26981928 | + | - |
| Nipah | Bangladesh | 2016 | Hegde ST, et al. Investigating Rare Risk Factors for Nipah Virus in Bangladesh: 2001-2012.  PMID: 27738775 | + | - |
| Nipah | Bangladesh | 2016 | Chakraborty A, et al. Evolving epidemiology of Nipah virus infection in Bangladesh: Evidence from outbreaks during 2010-2011  PMID: 26122675 | + | - |
| Nipah | Bangladesh | 2018 | Hassan MZ, et al. Nipah virus contamination of hospital surfaces during outbreaks, Bangladesh, 2013-2014  PMID: 29260663 | + | + |
| Nipah | Bangladesh | 2019 | Nikolay B, et al. Transmission of Nipah virus – 14 years of investigations in Bangladesh  PMID: 31067370 | + | + |
| Nipah | India | 2006 | Chadha MS, et al Nipah virus-associated encephalitis outbreak, Siliguri, India  PMID: 16494748 | + | + |
| Nipah | India | 2006 | Harit AK, et al. Nipah/Hendra virus outbreak in Siliguri, West Bengal, India in 2001  PMID: 16783047 | + | + |
| Nipah | India | 2011 | Arankalle VA, et al. Genomic characterization of Nipah virus, West Bengal, India.  PMID: 21529409 | + | + |
| Nipah | India | 2018 | Thulaseedaran NKA, et al. Case series on the recent Nipah epidemic in Kerala  PMID: 31317711 | + | + |
| Nipah | India | 2018 | Arunkumar G, et al. Outbreak investigation of Nipah virus disease in Kerala, India, 2018  PMID: 30364984 | + | + |
| Nipah | India | 2018 | Arunkumar G, et al. Persistence of Nipah virus RNA in semen of survivor  PMID: 30590474 | - | + |
| Nipah | India | 2019 | Arunkumar G, et al. Adaptive immune responses in humans during Nipah virus acute and convalescent phases of infection  PMID: 30615097 | - | + |
| Nipah | India | 2019 | Yadav PD, et al. Nipah virus sequences from humans and bats during Nipah outbreak, Kerala, India, 2018  PMID: 31002049 | - | +  (from secondary cases) |
| Nipah | India | 2019 | Kumar CPG, et al. Infections among contacts of patients with Nipah virus, India  PMID: 31002050 | + | - |
| Nipah-like | Philippines | 2015 | Ching PKG, et al. Outbreak of henipavirus infection, Philippines, 2014  PMID: 25626011 | + | + |

## **Supplementary Table 3.** Studies published through May 2019 on animal henipavirus infections, by first author name, year of publication, and animal model (N=78)

We identified 78 animal studies with primary data on the amount or duration of viral shedding, encompassing 11 animal model types: non-human primate, ferret, hamster, horse, pig, mouse, cat, dog, guinea pig, rat, and shrew (Supplementary Table 3). Thirty-six studies investigated infections with NiV_M_, 21 with HeV, 6 with NiV_B_, 1 with Cedar virus[^22^](https://paperpile.com/c/8ecMQN/rfDo) and 1 with Mòjiāng virus[^23^](https://paperpile.com/c/8ecMQN/Ojav). Six studies directly compared animals infected with NiV_M_ and NiV_B_, five compared NiV_M_ and HeV, and one compared NiV_M_, NiV_B_ and HeV. The Nipah virus strains used in animal studies were derived from a case-patient from the 2004 Rajbari, Bangladesh outbreak[^19^](https://paperpile.com/c/8ecMQN/7tMR) (NiV_B_) and a case patient from 1999 Malaysia outbreak among pig farmers[^24^](https://paperpile.com/c/8ecMQN/vO8B) (NiV_M_). The Hendra virus strains used in animal studies were derived from infected horses from the 1994[^15^](https://paperpile.com/c/8ecMQN/R9Br) and 2008[^25^](https://paperpile.com/c/8ecMQN/AeJS) outbreaks in Brisbane, Australia.

| **First Author** | **PMID** | **Publication Year** | **Animal model** | **Virus investigated** | | | |
| --- | --- | --- | --- | --- | --- | --- | --- |
|  |  |  |  | **HeV** | **NiV_M_** | **NiV_B_** | **Other Virus** |
| Mire* | 31107231 | 2019 | African green monkey |  |  | x |  |
| Lo | 31142680 | 2019 | African green monkey |  |  | x |  |
| Hammoud | 30462637 | 2018 | African green monkey |  | x |  |  |
| Cong | 28388650 | 2017 | African green monkey |  | x |  |  |
| Mire* | 27484128 | 2016 | African green monkey |  | x | x |  |
| Johnston | 25706617 | 2015 | African green monkey |  | x |  |  |
| Prescott | 25865472 | 2015 | African green monkey |  | x |  |  |
| Geisbert* | 24964990 | 2014 | African green monkey |  | x |  |  |
| Mire* | 24522928 | 2014 | African green monkey | x |  |  |  |
| Yoneda | 23516477 | 2013 | African green monkey |  | x |  |  |
| Bossart | 22875827 | 2012 | African green monkey |  | x |  |  |
| Bossart | 22013123 | 2011 | African green monkey | x |  |  |  |
| Geisbert | 20502528 | 2010 | African green monkey |  | x |  |  |
| Rockx | 20660198 | 2010 | African green monkey | x |  |  |  |
| Alimonti | 25059478 | 2014 | Cynomolgus macaques | x | x |  |  |
| Marianneu | 20202432 | 2010 | Squirrel monkey |  | x |  |  |
| Leon* | 29538374 | 2018 | Ferret | x | x | x |  |
| Clayton | 27341030 | 2016 | Ferret |  | x | x |  |
| Satterfield | 27147733 | 2016 | Ferret |  | x |  |  |
| Satterfield | 26105519 | 2015 | Ferret |  | x |  |  |
| Marsh | 23521919 | 2013 | Ferret | x |  |  |  |
| Mire | 24330654 | 2013 | Ferret |  | x |  |  |
| Pallister | 23867060 | 2013 | Ferret |  |  | x |  |
| Clayton* | 23171621 | 2012 | Ferret |  | x | x |  |
| Pallister* | 21689706 | 2011 | Ferret | x |  |  |  |
| Bossart* | 19888339 | 2009 | Ferret |  | x |  |  |
| Pallister | 19759137 | 2009 | Ferret |  | x |  |  |
| Schountz | 30909389 | 2019 | Syrian hamster |  |  |  | Cedar virus |
| Dawes | 29765101 | 2018 | Syrian hamster |  | x |  |  |
| Walpita | 29263876 | 2017 | Syrian hamster |  | x |  |  |
| Baseler | 27812087 | 2016 | Syrian hamster |  | x | x |  |
| Borisevich | 26357909 | 2016 | Syrian hamster | x | x |  |  |
| DeBuysscher | 28706736 | 2016 | Syrian hamster |  | x |  |  |
| Guillaume-Vasselin | 29263849 | 2016 | Hamster | x |  |  |  |
| DeBuysscher | 24631094 | 2014 | Syrian hamster |  | x |  |  |
| de Wit | 24626480 | 2014 | Syrian hamster |  |  | x |  |
| Lo | 24184127 | 2014 | Syrian hamster |  | x |  |  |
| DeBuysscher | 23342177 | 2013 | Syrian hamster |  | x | x |  |
| Ploquin | 23175762 | 2013 | Syrian hamster | x | x |  |  |
| Munster | 23071900 | 2012 | Syrian hamster |  | x |  |  |
| de Wit | 22180802 | 2011 | Syrian hamster |  | x |  |  |
| Mathieu | 21593145 | 2011 | Syrian hamster |  | x |  |  |
| Rockx | 21593160 | 2011 | Syrian hamster | x | x |  |  |
| Guillaume | 19328514 | 2009 | Syrian hamster | x |  |  |  |
| Georges-Courbot | 16641448 | 2006 | Syrian hamster |  | x |  |  |
| Guillaume | 14694115 | 2004 | Syrian hamster |  | x |  |  |
| Wong | 14578210 | 2003 | Syrian hamster |  | x |  |  |
| Cowled | 28785041 | 2017 | Horse | x |  |  |  |
| Ball | 24730376 | 2014 | Horse | x |  |  |  |
| Middleton | 24572697 | 2014 | Horse | x |  |  |  |
| Marsh | 22172152 | 2011 | Horse | x |  |  |  |
| Field | 20113576 | 2010 | Horse | x |  |  |  |
| Hooper | 10840579 | 2000 | Horse | x |  |  |  |
| Williamson | 9972433 | 1998 | Horse, Cat | x |  |  |  |
| Kasloff | 30914663 | 2019 | Landrace pig |  |  | x |  |
| Pickering | 27544586 | 2016 | Landrace pig | x | x |  |  |
| Li | 20167195 | 2010 | Guinea pig, Landrace pig, Gottingen minipig | x |  |  |  |
| Berhane | 18405339 | 2008 | Landrace pig |  | x |  |  |
| Weingartl | 16873250 | 2006 | Landrace pig |  | x |  |  |
| Weingartl | 15919907 | 2005 | Landrace pig |  | x |  |  |
| AbuBakar | 15663869 | 2004 | Pig |  | x |  |  |
| Tanimura | 15276859 | 2004 | Pig |  | x |  |  |
| Middleton | 11945001 | 2002 | Pig, Domestic shorthair cat |  | x |  |  |
| Escaffre | 28539439 | 2017 | Mouse (NOD-SCID/ɣc(null)) |  |  | x |  |
| Dups | 28388650 | 2014 | Mouse (BALB/c, C57BL/6) |  | x | x |  |
| Valbuena | 24699832 | 2014 | Mouse (NOD-SCID/ɣc(null)) |  | x |  |  |
| Dhondt | 23089589 | 2013 | Mouse (C57BL/6, IFNAR-KO) | x | x |  |  |
| Dups | 22808132 | 2012 | Mouse (BALB/c, C57BL/6) | x |  |  |  |
| Wu | 24865545 | 2014 | Buff-breasted rat, Musk shrew |  |  |  | Mòjiāng  virus |
| McEachern | 18556094 | 2008 | Domestic shorthair cat |  | x |  |  |
| Mungall | 17703410 | 2007 | Domestic shorthair cat fetus |  | x |  |  |
| Mungall | 17005664 | 2006 | Domestic shorthair cat |  | x |  |  |
| Middleton | 28124415 | 2017 | Beagle dog, Ferrets | x |  |  |  |
| Halima | 26536509 | 2015 | Terrier dog | x |  |  |  |
| Kirkland | 26583697 | 2015 | Fox terrier dog | x |  |  |  |
| Torres-Velez | 18587107 | 2008 | Guinea pig |  | x |  |  |
| Williamson | 11437503 | 2001 | Guinea pig | x |  |  |  |
| Williamson | 10684689 | 1999 | Guinea pig | x |  |  |  |
| *included in comparison analysis    ** PFU converted to TCID50 (PFU/0.7 = TCID_50_) | | | | |  |  |  |
|  |  |  |  |  |  |  |  |

**Supplementary information on ferret studies**

Four ferret studies, including 21 animals [^30–33^](https://paperpile.com/c/8ecMQN/hJlb+NHs5+WiTB+kDO3), were included in the analysis. We were unable to compare virus quantities across studies given a lack of comparable individual-level measurements of virus quantity in the published literature. However, 2 studies directly compared virus quantities at the group-level. Leon and colleagues[^31^](https://paperpile.com/c/8ecMQN/NHs5) inoculated groups of 4 ferrets intranasally with 5,000 50% tissue culture infectious dose (TCID_50_, approximately 3,500 PFU) of NiV_B_, NiV_M_, and HeV. On day 5 post inoculation, animals inoculated with NiV_B_ had the lowest mean virus quantity in nasal samples and animals inoculated with HeV had the highest mean virus quantity in oral samples. Animals inoculated with NiV_M_ had the lowest virus quantity in oral samples on days 6 and 7 post inoculation[^31^](https://paperpile.com/c/8ecMQN/NHs5). Clayton et al[^30^](https://paperpile.com/c/8ecMQN/hJlb) oronasally inoculated 8 ferrets with NiV_B_ and 7 ferrets with NiV_M_, all with 5,000 TCID_50_. They found no statistically significant difference in virus quantity over time in nasal samples (1 to 8 days post inoculation) but observed approximately 10-fold higher virus quantity in oral samples on days 5-6 and days 7-8 post inoculation among ferrets inoculated with NiV_B_. All but one animal in this study had culturable virus in their oral samples on the day of euthanasia.

Although we were unable to make individual-level comparisons of virus quantity, 3 of the ferret studies provided individual-level data on viral shedding duration across virus strains[^30,32,33^](https://paperpile.com/c/8ecMQN/hJlb+WiTB+kDO3). In all, 21 animals were inoculated with 5,000 TCID_50_ with 3 different henipavirus strains: 8 with NiV_B_, 11 with NiV_M_, and 2 with HeV. Inconsistent timing and frequency of sampling over the course of infection led to difficulties in comparing time to viral shedding onset and duration of viral shedding between virus strains (Supplementary Figure 5). However, once an animal began oral shedding, all additional samples had evidence of shedding until the day of euthanasia for all animals studied. The time point for euthanasia was based on severity of clinical signs. There were no notable differences in time from inoculation to euthanasia across strains: NiV_B_ (mean, range: 7.6, 7-9), NIV_M_ (8, 5-10), and HeV (7, 7-7). Daily individual-level clinical signs were not reported in any of four studies. Two animals infected with HeV had oral samples investigated for culturable virus on the day of euthanasia; 1 animal had cultural virus in their sample.[^32^](https://paperpile.com/c/8ecMQN/WiTB)

**Supplementary Figure 5.** Comparing duration of oral viral shedding, respiratory signs, and timing of humane endpoints among ferrets (N=21) infected with Hendra virus, Nipah virus Bangladesh (NiV_B_), or Nipah virus Malaysia (NiV_M_), by study.

**
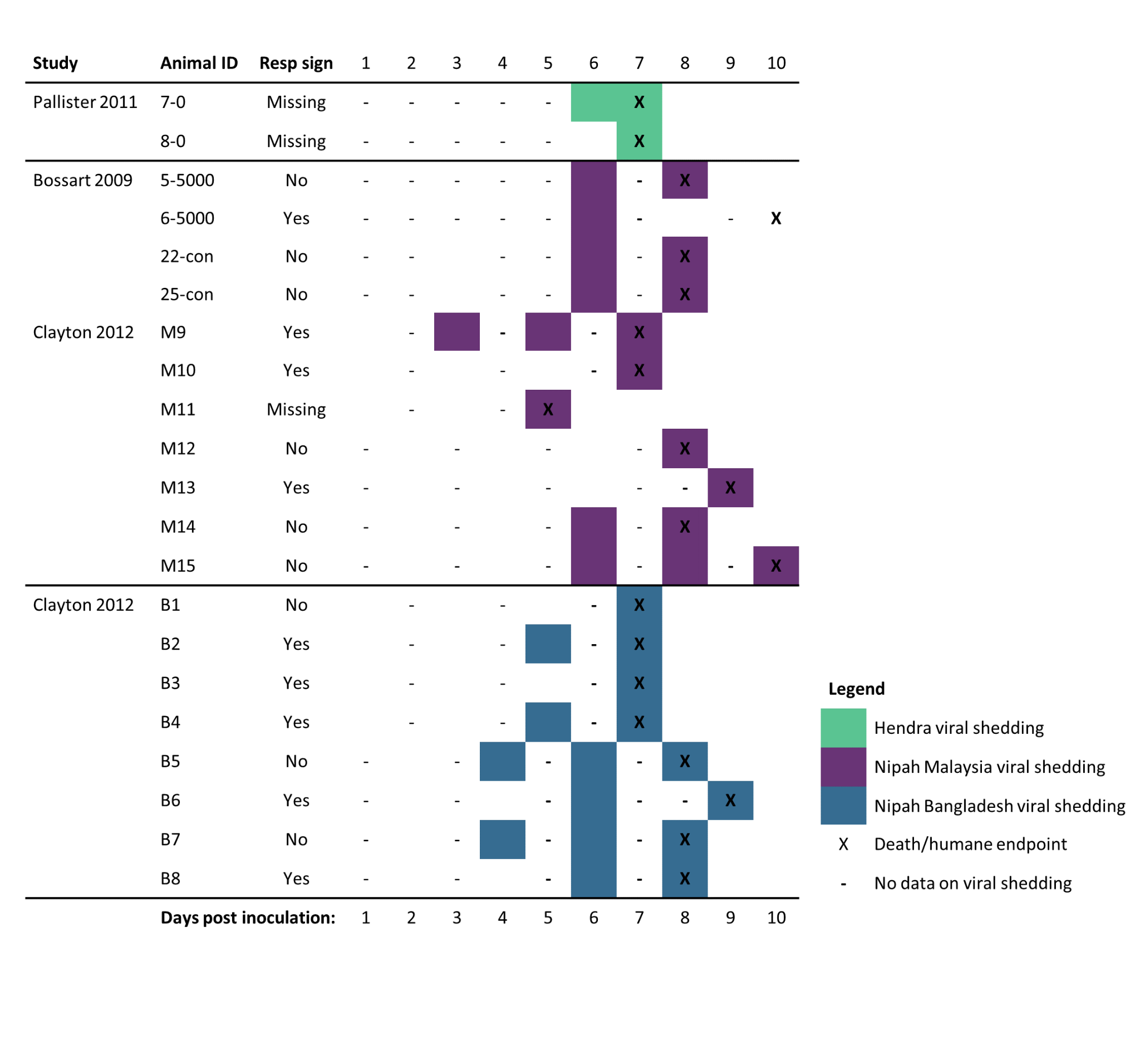
**

**Supplementary suggestions on outbreak case reporting**

Outbreak investigations should report as much granularity as possible on the route of exposure, timeline of infection and symptoms (e.g. incubation period), paired with serial oral/respiratory and blood specimens with viral load (or Ct values as an indicator), evaluation of other potentially infectious body fluids (e.g. tears, semen, vaginal fluids), case fatality ratio (CFR), timing of death, proportion of transmitters, geographic distribution, spillover frequency, and cluster size[^38^](https://paperpile.com/c/8ecMQN/HbEY). The potential for transmission can be quantified by then establishing such measures as the secondary attack rate, duration of viral shedding, quantity of virus shed, and clinical signs and symptoms that promote transmission of the virus to others. Therefore, publishing detailed line lists from outbreaks could help to build a global database of henipavirus transmission that could be leveraged to improve our collective understanding over time.
